# Safety and feasibility of a multimodal approach for orchestra musicians with playing-related musculoskeletal disorders (PRMDs)

**DOI:** 10.1007/s00508-025-02566-y

**Published:** 2025-07-28

**Authors:** Paul Emmerich Krumpoeck, Gerold Ebenbichler, Christina Knosp, Ricarda-Samantha Roiger-Simek, Nicoletta Margreiter-Neuwirth, Wolfgang Neuwirth, Gregor Kasprian, Karl-Heinz Nenning, Victor Schmidbauer, Emir Benca, Fritz Sterz

**Affiliations:** 1 Department of Emergency Medicine, Medical University of Vienna, Währinger Gürtel 18–20, 1090 Vienna, Austria; 2 Department of Physical Medicine, Rehabilitation and Occupational Medicine, Medical University of Vienna, Vienna, Austria; 3 Medical University of Vienna, Vienna, Austria; 4 Division of Neuroradiology and Musculoskeletal Radiology, Department of Radiology, Medical University of Vienna, Vienna, Austria; 5 Division of Biomedical Imaging and Image-guided Therapy, Computational Imaging Research Lab, Department of Radiology, Medical University of Vienna, Vienna, Austria; 6 Center for Biomedical Imaging and Neuromodulation, Nathan Kline Institute, Orangeburg, NY USA; 7 Department of Orthopedics and Trauma-Surgery, Medical University of Vienna, Vienna, Austria; 8 Comprehensive Center for Musculoskeletal Disorders (CCMSD), Medical University of Vienna, Vienna, Austria

**Keywords:** Occupational diseases, Transcranial direct current stimulation, Physical therapy, Counseling, Magnetic resonance imaging

## Abstract

**Introduction:**

Orchestra musicians frequently experience painful playing-related musculoskeletal disorders (PRMDs) yet often lack access to effective specialized treatment. This feasibility study aimed to establish proof-of-concept for a novel, multimodal treatment regimen and to explore potential diagnostic tools for PRMDs.

**Methods:**

Musicians from the Orchestra Academy of the Vienna Philharmonic Orchestra participated in different interventions from a therapeutic and diagnostic protocol. The therapeutic part encompassed self-administered transcranial direct current stimulation (tDCS) sessions targeting the primary motor cortex with tailored physiotherapeutic exercises and psychological coaching. Separate diagnostic interventions included comprehensive physiotherapeutic and psychological assessments, pain questionnaires, and the acquisition of magnetic resonance imaging and 3D motion capture data. The feasibility of these methods was thoroughly evaluated through safety questionnaires, completion checklists, direct observation by the investigators, and detailed participant feedback.

**Results:**

The therapeutic tDCS sessions with concurrent physiotherapeutic exercises were completed by 2 participants across all 10 scheduled sessions. Mild to moderate tingling/burning sensations during tDCS sessions were reported in 3 of 10 sessions (30%), and electrode connectivity issues occurred in 3 of 10 sessions (30%), which participants could resolve independently. All seven participants engaged in various diagnostic assessments. The novel pain assessment questionnaire was completed by four participants in under 5 min, with reported pain intensities ranging from 0–5 on a 0–10 scale, most frequently in the neck, wrist/hand, and upper and lower back. Functional magnetic resonance imaging during simulated instrument playing revealed discernible activation patterns, including bilateral primary motor cortex activation, and 3D motion capture provided detailed kinematic data from a violinist.

**Discussion:**

This study provides initial evidence for the feasibility and safety of a combined treatment approach (tDCS, physiotherapy, psychological support) for musicians suffering from PRMDs. Furthermore, the results encourage further exploration of advanced imaging and motion capture techniques as potential diagnostic and monitoring tools. These findings support conducting a larger scale, randomized clinical trial to investigate the efficacy of these approaches.

**Supplementary Information:**

The online version of this article (10.1007/s00508-025-02566-y) contains supplementary material, which is available to authorized users.

## Introduction

In the USA alone there are about 2200 orchestras consisting of about 220,000 musicians (40,000 professional), which give more than 25,000 performances per year combined [1]. In most cases, top performing orchestra musicians start playing their instrument at a very early age with continuing lessons, competitions, and touring. This predisposes them to a “no pain, no gain” mindset, prioritizing professional success over physical and mental health [2], neglecting the physical and emotional impact of their typically demanding schedule of practicing and performances [3, 4]. All of this facilitates the development of a variety of very different diseases, such as focal task-specific dystonia, music performance anxiety, depression, eating disorders, and playing-related musculoskeletal disorders (PRMDs) [5]. Also, many musicians are reluctant to do physical exercises prior to performances or to consult their teachers, let alone healthcare providers, to not be perceived as less talented or competent. Therefore, these diseases can often remain ignored and thus untreated [6].

With prevalences as high as 94.8% and often poor outcomes, PRMDs and other disorders linked to performance are still severely underrecognized clinical problems [7]. Despite sporadic performing arts medicine initiatives, there is no widely accepted medical coaching strategy accessible for performers in many parts of the world [8]. There is an urgent need for methods that can help investigate and treat PRMDs and further support access to professional occupational care.

A complex motor task such as playing a musical instrument must be learned through years of practice. It has been shown that musical training modulates certain brain areas through mechanisms of neuroplasticity, especially those related to auditory and sensorimotor networks, regions distributed throughout the brain such as the intraparietal sulcus, superior temporal gyrus, insula-based networks, and more [9, 10]. Transcranial direct current stimulation (tDCS) is a noninvasive brain stimulation technique, which modulates neural plasticity through a weak electric current that is delivered by two or more conductive electrodes placed on the scalp [11]. It is noninvasive, relatively inexpensive, safe, and easy to administer, making it a promising tool for therapy in a wide variety of fields. Clinical investigations have been conducted for many different diseases, among which are depression, chronic pain, and post-stroke motor rehabilitation [11].

The ability of tDCS to improve learning of new motor patterns through stimulation of the primary motor cortex (M1) has been studied extensively and was even observed in musicians, although the effects of stimulation seem to correlate negatively with the level of skill already acquired [12, 13]. Paired with physiotherapeutic exercises, this could help musicians suffering from PRMDs with retraining of their motor skills to alter especially stressful motion patterns, thereby alleviating the pain. In addition, it could also potentially contribute to preventing healthy musicians from incurring PRMDs in the first place.

To evaluate a musician’s pain situation, there are several questionnaires established in clinical practice [14, 15]. In contrast to these subjective assessments, the underlying alterations in a musician’s neuromuscular circuits and motor patterns could also be quantified with objective measurements. Structural magnetic resonance imaging (MRI) and blood oxygen level dependent (BOLD) functional MRI (fMRI) of the brain have been used to compare musicians and non-musicians, finding differences in activity in many regions (e.g., posterior superior temporal gyrus) [16, 17] as well as shared networks between auditory and motor processing [18, 19]. Furthermore, motion capture can accurately monitor even very complex movements, such as that of the hand, which makes it very useful for objectively evaluating musicians’ motor patterns, including postural and neuromuscular disorders [20, 21].

The objective of this study was to provide a proof of concept of a novel treatment plan for PRMDs. This plan combines tDCS with physiotherapeutic exercises and psychological coaching, aiming to aid musicians in retraining painful motion patterns and dealing with pain-related consequences. Furthermore, we also investigated diagnostic tools that could help evaluate the utility of this treatment plan, i.e., a novel pain questionnaire, medical imaging methods, and motion capture techniques. To this end, a preliminary trial was conducted to demonstrate the safety and feasibility of these interventions.

## Material and methods

This study was conducted under approval of the ethics committee of the Medical University of Vienna (ethics committee number 1111/2021, approved 26.05.2021, extended 17.05.2022). Participants were recruited via e‑mail from the Orchestra Academy of the Vienna Philharmonic Orchestra and provided written informed consent. They could participate in their choice of the interventions described in the following paragraphs, although the number of participants per intervention was limited for practical reasons. An overview of the trials chosen by each participant can be found in Supplementary Table 1.

### Transcranial direct current stimulation

For the tDCS intervention, participants self-administered 5 sessions each of 20 min with an intensity of 2.0 mA (current density: 0.07 mA/cm^2^) to the M1 delivered by the commercial Halo Sport 2 device (Halo Neuroscience, Flow Neuroscience, Malmö, Sweden). In this model, the tDCS technology is built into a pair of audio headphones with adjustable size. The electrodes are fixed inside a removable strap on the side of the headband facing the scalp and, when put on, lie directly above the M1. The electrode positions, based on the International 10-20 system for locating scalp sites, are CZ (at the vertex) for the cathodal electrode (size: 6 cm × 4 cm) and C5/C6 (overlying the motor cortices) for the anodal electrodes (size: 4 cm × 4 cm) [22]. This simple handling enabled participants to do the sessions at home on 5 days of 1 week, with 2 rest days that could be chosen freely, albeit not consecutively.

### Physiotherapeutic exercise program and assessment

During the sessions, the participants completed a physiotherapeutic exercise program, which was tailored to each participant’s individual symptoms and requirements. The purpose is to help optimize strength, coordination and endurance of the muscles, thereby facilitating an ergonomic posture on the instrument. It was based on the results of the initial physiotherapeutic assessment, which took place before the tDCS trials and was intended to identify (non)physiological postures and motion patterns. It consisted of a clinical examination that included the modified Upper Quarter Y‑Balance Test (mUQYBT) [23], the Closed Kinetic Chain Upper Extremity Speed Test (CKCUEST) [24], the One-Arm Line Hopping Test, a modified version of the One-Arm Hop Test [25] and the QuickDASH questionnaire [26]. The exercise program started concurrently to the start of tDCS with a short warm-up, after which a combination of five coordination and strength exercises followed. The duration of each daily program was approximately 22–25 min, so that exercises could be performed during the entire tDCS session. Afterwards, participants were encouraged to practice their instrument until 1h after the stimulation had ended. When the participants received the programs, each exercise was explained, and the proper technique was discussed with and demonstrated to the participants, until they had no further questions and stated that they knew how to execute their tasks. They were asked to document the completion of the tDCS sessions and any issues in a tDCS and Physio exercise checklist (see Table 1), while adverse events were assessed in a separate tDCS safety questionnaire (see supplementary information).

**Table 1:** tDCS and Physio exercise checklist. Participants received this checklist to document the completion of their programs and any problems that might occur. They handed in the checklist after the week of stimulations

Date	tDCS	Physio-exercises
Day 1	Completed: yes □—no □ Problems: yes □—no □ If yes, which:	Completed: yes □—no □ Problems: yes □—no □ If yes, which:
Day 2	Completed: yes □—no □ Problems: yes □—no □ If yes, which:	Completed: yes □—no □ Problems: yes □—no □ If yes, which:
Day 3	Completed: yes □—no □ Problems: yes □—no □ If yes, which:	Completed: yes □—no □ Problems: yes □—no □ If yes, which:
Day 4	Completed: yes □—no □ Problems: yes □—no □ If yes, which:	Completed: yes □—no □ Problems: yes □—no □ If yes, which:
Day 5	Completed: yes □—no □ Problems: yes □—no □ If yes, which:	Completed: yes □—no □ Problems: yes □—no □ If yes, which:

*tDCS* transcranial direct current stimulation

### Psychological assessment

Additionally, an initial psychological assessment session was held, in which the ability to learn as well as cognitive processing speed, planning ability, and inhibition (i.e., the ability to suppress an unwanted reaction) were examined through five different established tests (see supplementary information). All tests were acquired from Schuhfried GmbH (Mödling, Austria) and conducted as described in their respective manuals. Following the completion of the neuropsychological tests, the participants’ results were presented to them by the psychologist and discussed further. The results of this assessment are intended to serve as a basis to evaluate the subject’s psychological status and for the individual adaptation of cognitive and behavioral therapies (CBTs). Due to the nature of CBTs, the weekly coaching sessions vary greatly between different musicians and require repeated adjustments over time to be properly executed. As this would not have been possible within the scope of a feasibility trial, and because CBTs are well-established in clinical practice, no such sessions were done in this study.

### Pain assessment questionnaire

As a possible primary outcome measure for a larger study with this multimodal treatment regimen, a pain assessment questionnaire (see Table 2) was designed, based on the Nordic Musculoskeletal Questionnaire (NMQ) [14], the DASH [15] and the Brief Illness Perception Questionnaire (BIPQ) [27]. It was developed in order to provide a shorter but still comprehensive questionnaire to reduce participants’ effort when completing the questionnaire repeatedly in potential future studies. After the week of stimulations, the participants filled out both the pain assessment questionnaire and the tDCS safety questionnaire and handed them in alongside the tDCS and Physio exercise checklist.

**Table 2:** Pain assessment questionnaire. Participants filled out this questionnaire at their visit following the week of stimulations and exercises

**How much PAIN have you had IN THE PAST MONTH?**		
*On a scale of 0 to 10 (where zero represents “no pain” and 10 represents “severe pain”), please record the number below*	-	*No pain (0) — Severe pain (10)*
​ 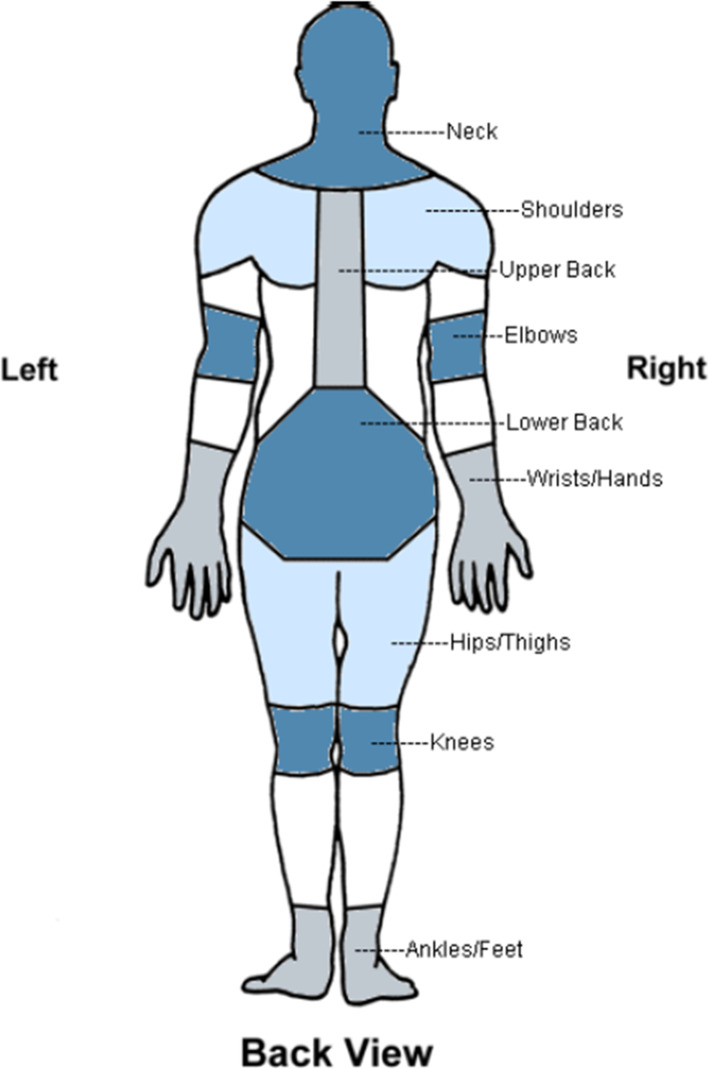	*Neck*	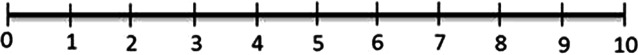
	*Shoulders*	
	Right shoulder	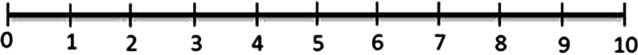
	Left shoulder	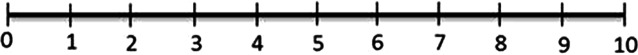
	Both shoulders	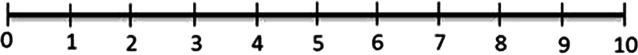
	*Elbows*	
	Right elbow	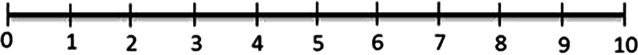
	Left elbow	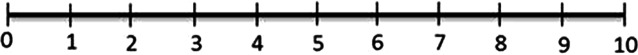
	Both elbows	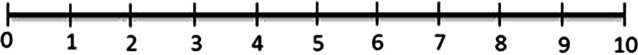
	*Wrists/hands*	
	Right wrist/hand	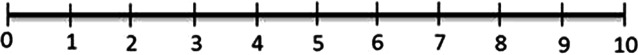
	Left wrist/hand	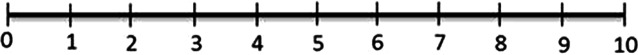
	Both wrists/hands	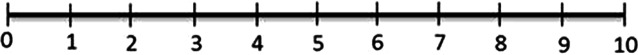
	*Upper back*	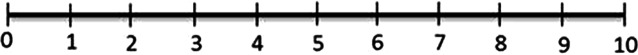
	*Lower back*	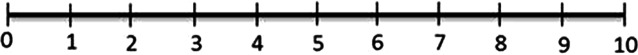
	*One or both hips/thighs*	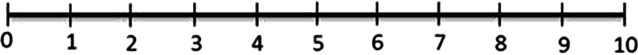
	*One or both nees*	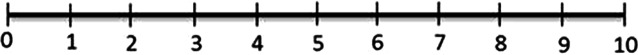
*Please describe your physical ability in the past week. Did you have any difficulty…*		*No difficulty (0) *—* Unable (10)*
… using your usual technique for playing your instrument?		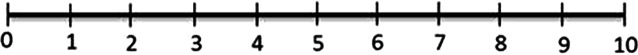
… playing your musical instrument because of arm or shoulder pain?		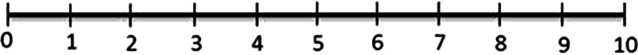
… playing your musical instrument as well as you would like?		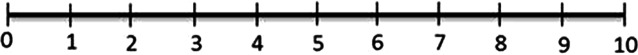
… completing all of your practice exercises in the scheduled time?		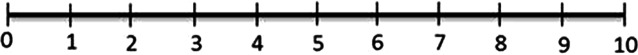

### Imaging

The imaging protocol had a total duration of approximately 45 min and included a structural T1 acquisition (5 min), diffusion tensor imaging (DTI; 8 min), and resting state fMRI (15 min), during which the musician was instructed to lie in the scanner at rest. Then, three task-based fMRI acquisitions (5 min each) were done, in which the participants pretended to play a given piece of music without moving and then additionally moved their fingers on a wooden fingerboard as if they were playing. In the last task-based fMRI acquisition, there were alternating blocks of activation through mental exercise and baseline condition for 30 s each. Functional activity, functional connectivity (based on BOLD fMRI), structural connectivity (based on DTI), and cortical thickness/configuration patterns (based on high-resolution T1-weighted images) were assessed for each task.

### 3D motion capture

For the 3D motion capture analysis, the opto-electronic tracking system SMART‑E (BTS S.p.A., Milano, Italy) was used to assess the kinematics in a single musician. The system consists of four cameras, which record the position of light-reflecting markers in 3D space. The angles of the wrist, elbow, and shoulder, as well as their mean absolute deviation, were obtained at a sampling frequency of 125 Hz for 2 performances. The kinematic data were processed using the system’s own software, Smart Analyzer 1.10.

## Results

A total of seven musicians participated in the study, with each musician completing 1–3 of the different parts of the study. Table 3 shows descriptive statistical parameters of the study population and lists the interventions that were chosen by each participant. The data underlying the results described here as well as more descriptive and outcome data, can be found in the supplementary information.

**Table 3:** Descriptive statistics of study participants. Various parameters as well as the selected interventions displayed separately for each individual participant

Participant ID	Age (years)	Sex	BMI	Instrument	Age Instr	Years Instr	Most painful region	Pain severity (×/10)	Interventions selected
1	31	M	22.0	Violin	8	23	n. a.	n. a.	Imaging
2	27	F	23.2	Violin	5	22	n. a.	n. a.	Imaging
3	20	M	20.9	Violin	4	16	n. a.	n. a.	Motion capture
4	27	M	19.1	Viola	5	22	Neck, shoulders, wrist	1.0	tDCS + Physio
5	25	F	22.6	Flute	5	19	Neck	5.0	Physio
6	25	M	24.2	Trombone	7	18	Neck, upper back	4.0	tDCS + Physio + Psychological assessment
7	23	M	25.4	Trumpet	7	16	Wrist, lower back	3.0	Physio + Psychological assessment
Average	25.4	–	22.5	–	5.9	19.4	–	3.25	–

*Age Instr* age of first instrument practicing, *Years Instr* years of instrument playing, *BMI* body mass index

### Transcranial direct current stimulation

Of the participants two engaged in tDCS and both completed five sessions each. No issues that hindered completion were encountered for all 10 tDCS sessions together with their concurrent physiotherapeutic exercises. Both participants stated in their visits that the device was easy to administer. No severe adverse events of tDCS were reported but there were some adverse events and other issues, which are listed in Table 4. A mild to moderate tingling/burning sensation on the scalp was reported by 1 participant for 3 sessions (3/10 sessions in total), while the most frequent technical issue was a lack of electrode connectivity, which also occurred in 3/10 sessions. As the participants had been informed about this issue, they were able to solve this problem independently and were thus able to complete these sessions as well.

**Table 4:** Absolute (%) frequency of issues with tDCS and the physiotherapeutic exercises. Number of occurrences of each issue displayed separately for participants 1 and 2 as well as the total number among all 10 sessions with relative fractions in brackets (%).

Issue	Participant 1	Participant 2	Total (%)
Connectivity	2	1	3 (30)
Lack of moisture	1	0	1 (10)
Falling off	1	0	1 (10)
Tingling/burning	3	0	3 (30)

### Physiotherapeutic exercise program and assessment

Table 5 gives an example of the physiotherapeutic exercise program, which was designed individually for one of the participants based on his performance in the initial physiotherapeutic evaluation. Both participants documented the completion of the exercises in the checklist and reported no trouble following their programs or practicing their instruments afterwards. The physiotherapeutic assessment, including the general physiotherapeutic evaluation and the execution of the mUQYBT, the CKCUEST, and the QuickDASH questionnaire, proceeded without issues. The one-arm line hopping test was completed by two participants, as the third suffered from a ganglion cyst on his right wrist. The dynamic weight put on the diseased wrist would have caused pain and possibly worsened the outcome of the condition, which is why this test was omitted for this participant.

**Table 5:** Example 1‑week exercise program. This program of physiotherapeutic exercises was individually designed for one of the participants and done during the tDCS sessions

Exercise	Description
*Warm-up exercises*	
Shoulder, elbow, and arm movements while standing	10 times backwards
	10 times in opposite directions
Cervical spine rotation + nodding the head	5 times each looking to the left, middle, and right, 3 times nodding the head in the three positions mentioned above while sitting
Thoracic spine rotation in combination with cervical and lumbar spine rotation (global rotation)	10 times isolated rotation of the thoracic spine, 10 times global rotation
Lumbar spine mobilization	Lumbar spine mobilization while sitting
Cervical stabilizers	Quick head nodding and shaking movements alternately while sitting and standing
*Strengthening exercises*	
Serratus push (serratus anterior muscle)	Dorsal position in closed chain
Shoulder shrugs (Trapezius pars descendens muscle)	In combination with deep breathing (contract—relax)
Chopping exercise (sitting position with 90° flexion of the shoulders: movement: very small and quick movements of the arms)	While sitting
Transversus abdominis muscle	Dorsal position with well positioned legs (approx. 120° knee flexion)
Muscle chain ventral + dorsal PNF D1	With no weight alternately while sitting and standing for every training session
Muscle chain ventral + dorsal PNF D2	With no weight alternately while sitting and standing for every session

*PNF D1/D2* proprioceptive neuromuscular facilitation (exercise), diagonal pattern 1/2

### Psychological assessment

In the psychological assessment, both participants were able to complete all five tests without assistance using a computer. Neither had any questions regarding their tasks, although the psychologist was present to provide further assistance if needed. Both participants reported a satisfactory experience with the tasks and the achieved results, and neither stated to be particularly worn out by the assessments.

### Pain assessment questionnaire

Regarding the novel diagnostic tools, all four participants who filled out the pain assessment questionnaire understood it without any further clarification and were able to complete it in under 5 min. As detailed in Table 6, reported pain intensities ranged from 0–5 points on a scale of 0–10 points, with the most frequent locations of pain being the neck, the wrist/hand, and the upper and lower back. While pain was reported by 3 of 4 participants in all these regions, no region was described as painful by all 4 participants.

**Table 6:** Absolute frequency and mean (SD) severity of pain in different body regions reported in the pain assessment questionnaire (*n* = 4 musicians)

Body region	Frequency	Severity
Neck	3	2.5 (2.1)
Shoulders	2	0.9 (1.2)
Elbows	0	0 (0)
Wrists/hands	3	0.6 (0.5)
Upper back	3	2.0 (1.6)
Lower back	3	2.0 (1.2)
Hips/thighs	0	0 (0)
Knees	1	0.3 (0.4)

*SD* Standard deviation

### Imaging

**Fig. 1:**
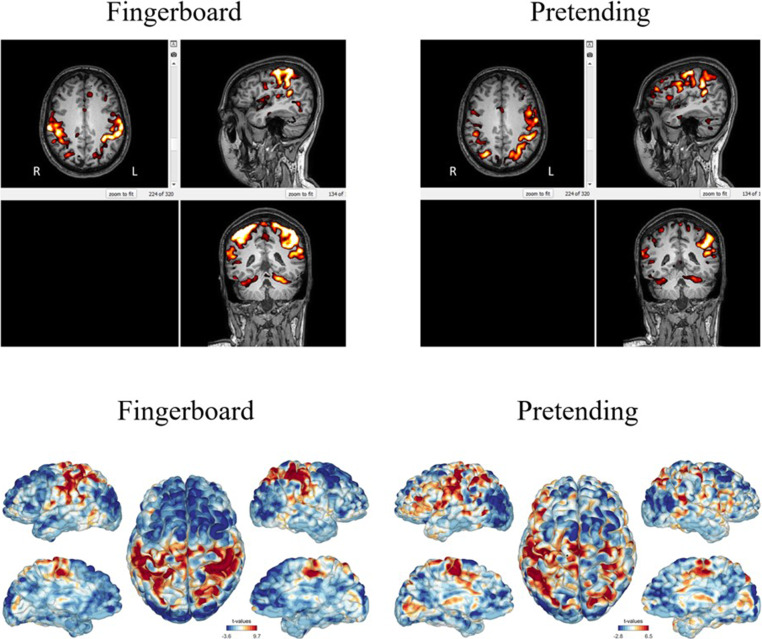
Task-based BOLD fMRI activation in a single participant. The figure displays Blood Oxygen Level-Dependent (BOLD) signal changes during two simulated music-playing tasks. (Left) The “Fingerboard” task involved finger movements on a wooden fingerboard. (Right) The “Pretending” task involved only motor imagery of playing a musical piece. The top row shows statistical activation maps (axial, sagittal, and coronal views) overlaid on the participant’s T1-weighted anatomical scan while the bottom row displays the same activations projected onto a 3D cortical surface model. The color-coding indicates areas of significant BOLD signal increase (activation) in warm colors (red, yellow) and signal decrease (deactivation) in cool colors (blue) compared to a resting baseline. Both tasks elicited activation in sensorimotor networks, with discernible activation in the primary motor cortex (M1), particularly in the hand motor area, during the “Fingerboard” task

During the 45-min imaging protocol, participants regularly confirmed their well-being through the MRI scanner’s intercom system between the individual acquisitions. After the trials, both participants reported being able to perform all their exercises in the right order without disturbances within the MRI scanner. Fig. 1 shows the first two task-based fMRI acquisitions of one of the participants. They were acquired during the first 2 tasks of 5min each, in which the participant pretended to play a piece of music for 5 min (right) and additionally moved his fingers on a wooden fingerboard (left). Overall, activation patterns can be found in both participants. In the “fingerboard” exercises, participant 1 shows a clear activation of the right M1, specifically in the right hand motor area. This activation can be observed even more clearly in participant 2, where the activation of the hand knobs takes place not just in the right M1, but bilaterally. In the “pretend” exercise, participant 2 shows a similar activation pattern as in the “fingerboard” task, although the activation seems to be lateralized more to the left hemisphere. More acquisitions can be found in the supplementary information.

### 3D motion capture

**Fig. 2:**
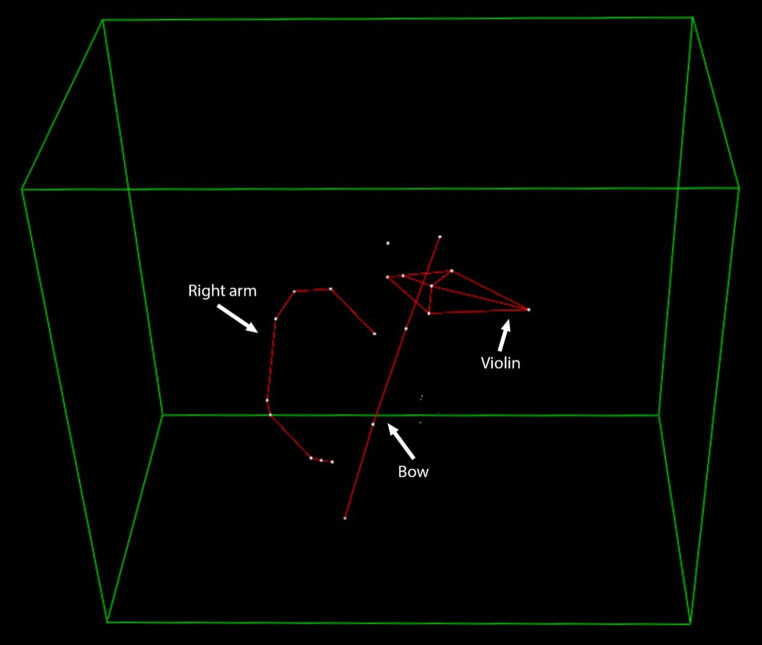
Kinematic model from 3D motion capture of a violinist. This image is a snapshot of a three-dimensional reconstruction generated from an opto-electronic motion capture system during a violin performance. The white dots represent the recorded positions of light-reflecting markers that were strategically placed on the musician’s upper body, the violin, and the bow. The red lines are computer-generated connections between these markers, creating a kinematic “stick-figure” model. This technique allows for the precise, quantitative analysis of movement, including the joint angles of the wrist, elbow, and shoulder, to objectively evaluate a musician’s motor patterns

In the 3D motion capture trial, the light-reflecting markers fixed with adhesive strips maintained strong contact with the skin but were light enough so that the musician had no problems concentrating on the performance. A sample of the raw data acquired for each joint angle continually recorded across two plays can be found in the supplementary information. It was used to generate a continuous 3D reconstruction of the violinist over time, of which a snapshot is depicted below in Fig. 2.

## Discussion

This feasibility study aimed to establish proof-of-concept for a novel, multimodal therapeutic regimen combining tDCS, physiotherapy, and psychological support, and to explore the feasibility of advanced diagnostic tools for PRMDs. We provide initial evidence for the feasibility and safety of the investigated therapeutic components in musicians. Participants completed self-administered tDCS sessions targeting the M1 concurrently with individualized physiotherapeutic exercise programs. These interventions were tolerated, with participants reporting only minor transient adverse effects, such as mild tingling sensations during tDCS. This incidence of mild adverse effects (3/10 sessions with tingling/burning) aligns with the existing literature on tDCS, where such effects are frequently reported and are generally not significantly different from sham stimulation [28]. The ability of musicians to self-administer tDCS at home with minimal issues, such as occasional electrode connectivity problems (a known technical aspect of some tDCS devices [22] that participants could independently resolve), supports the practicality and potential for broader application of this neuromodulatory technique as part of a comprehensive treatment plan. Furthermore, the initial psychological assessments were completed without difficulty, providing a foundation for incorporating tailored cognitive and behavioral therapies in future, more extensive trials. The completion of these therapeutic components, even concurrently by one participant, suggests that such an integrated approach is viable and not overly burdensome for musicians.

A second finding is the implementation and participant completion of a suite of diagnostic and assessment tools. The comprehensive physiotherapeutic evaluations, including functional tests and the QuickDASH questionnaire, were completed effectively. Moreover, the novel pain assessment questionnaire, designed for brevity and comprehensiveness, was readily understood and rapidly completed by participants. Notably, the pain distribution patterns identified using this new questionnaire, with the neck, wrist/hand and back being most frequently affected, are consistent with larger epidemiological studies on pain prevalence in musician populations [29, 30]. This congruence supports its potential utility for efficiently tracking pain in future studies. Beyond subjective and clinical assessments, this study also successfully evaluated the feasibility of advanced objective measures. During simulated instrument playing, fMRI revealed discernible activation patterns, including in the M1 (hand knob area), which is a key region for motor control and learning and known to be modulated by musical training [9, 10, 17, 19]. Similarly, 3D motion capture provided detailed kinematic data during performance. The successful acquisition of such data underscores the potential of these techniques to objectively quantify motor patterns and neural correlates of PRMDs, and to track changes in response to interventions.

This study adds to the existing literature by providing initial evidence for the feasibility of an integrated multimodal approach specifically for PRMDs in orchestra musicians. While tDCS has shown promise in motor rehabilitation and pain management [11] and its effects on motor learning in musicians have been explored [12, 13], its combination with structured physiotherapy and psychological assessment in this highly specialized population is novel. By demonstrating that musicians can manage self-administered tDCS alongside their practice and tailored exercises, this initial investigation offers a practical model for future interventions. The exploration of fMRI and 3D motion capture as complementary diagnostic tools in this specific context also contributes to the nascent field of applying advanced neuroimaging and biomechanical analysis to understand and manage performance-related disorders [16, 20, 21].

When interpreting these results, all conclusions regarding the safety and reliability of the many different tools and methods must be drawn cautiously due to the small sample size (*n* = 7). As this was merely a feasibility pilot trial that aimed for a proof of concept, a larger number of participants would have exceeded the scope of the study. Nevertheless, one participant easily completed all three therapeutic trials at the same time, alleviating the concern that doing all the trials at once could be too demanding.

Furthermore, our results are reliant on the musicians’ subjective statements, which are influenced by their personal opinions and feelings; however, this study is focused less on the specific results generated through the trials and more on the fundamental feasibility of conducting the trials in the first place. Therefore, although these statements are not as objective as results obtained through measurements, they are often the most important outcomes.

Moving forward, these promising findings support the need for a larger scale, randomized controlled clinical trial. Such a trial should aim to rigorously evaluate the efficacy of the combined treatment regimen in reducing pain, improving function and modifying maladaptive motor and neural patterns in musicians with PRMDs. Future research should also focus on optimizing the parameters of each intervention component, investigating the long-term effects of the treatment and further validating the novel pain questionnaire and the utility of fMRI and 3D motion capture as biomarkers of disease severity and treatment response. These objective measures could provide crucial insights into the neurophysiological and biomechanical mechanisms underlying PRMDs and their amelioration.

In conclusion, this feasibility study successfully demonstrated the safety and feasibility of a multimodal approach involving self-administered tDCS, physiotherapy and psychological support for orchestra musicians with PRMDs. Furthermore, multiple diagnostic tools, including a novel pain questionnaire, fMRI, and 3D motion capture, proved practical to implement. These findings lay a critical foundation for future trials aimed at establishing effective, evidence-based interventions to support the health and careers of musicians.

## Supplementary Information

Figures and Tables from the case studies.

## Data Availability

Aside from the data and other information reported in the “results” section, more data underlying the results, more descriptive data and other illustrations can be found in the supplementary information. Additional information on the study methodology as well as the entire raw data, will be made available by the authors upon reasonable request.
