# Supplementary material for: Safety and feasibility of a multimodal approach for orchestra musicians with playing-related musculoskeletal disorders (PRMDs)
Source: Wien Klin Wochenschr. 2025 Jul 28;138(1-2):38–47. doi: 10.1007/s00508-025-02566-y (PMC12830390; doi:10.1007/s00508-025-02566-y)
Supplement: Supplementary file 1 — Figures and Tables from the case studies. [file 508_2025_2566_MOESM1_ESM.docx]

Safety and feasibility of a multimodal approach for orchestra musicians with playing-related musculoskeletal disorders (PRMDs).
Supplementary Material

# Supplementary Figures

[Figure 1: Medical imaging protocol with acquisition types and durations.](#_Toc150020643)

[Figure 2: Percentile ranks achieved by participant 1 in the TOL-F. (TOL-F = Tower of London – Freiburg Version)](#_Toc150020644)

[Figure 3: Percentile ranks achieved by participant 2 in the TOL-F. (TOL-F = Tower of London – Freiburg Version)](#_Toc150020645)

[Figure 4: Percentile ranks with CIs achieved by participant 1 in the INHIB. (INHIB = Response Inhibition, CI = Confidence Interval)](#_Toc150020646)

[Figure 5: Percentile ranks with CIs achieved by participant 2 in the INHIB. (INHIB = Response Inhibition, CI = Confidence Interval)](#_Toc150020647)

[Figure 6: Task-based BOLD fMRI scans of participant 1.](#_Toc150020648)

[Figure 7: Task-based BOLD fMRI scans of participant 2.](#_Toc150020649)

[Figure 8: DTI Tractography of participant 1.](#_Toc150020650)

[Figure 9: DTI Tractography of participant 2.](#_Toc150020651)

[Figure 10: Wrist angle for two plays.](#_Toc150020652)

[Figure 11: Elbow angle for two plays.](#_Toc150020653)

[Figure 12: Shoulder angle for two plays.](#_Toc150020654)

[Figure 13: Absolute deviation for the wrist-, elbow-, and shoulder angle.](#_Toc150020655)

[Figure 14: 3D motion capture model reconstruction (front view).](#_Toc150020656)

# Supplementary Tables

Table 1: Pilot trials completed by each participant. (tDCS = transcranial Direct Current Stimulation, MI = Medical Imaging)

Table 2: Number of participants and total time (minutes) per pilot trial.

Table 3: Absolute frequency of issues with tDCS and the physiotherapeutic exercises.

Table 4: tDCS safety questionnaire.

Table 5: Answers to the "tDCS safety questionnaire" statements.

Table 6: Distances (cm) achieved by participant 1 in the mUQYBT. (mUQYBT = modified Upper Quarter Y-Balance Test)

Table 7: Distances (cm) achieved by participant 2 in the mUQYBT. (mUQYBT = modified Upper Quarter Y-Balance Test)

Table 8: Distances (cm) achieved by participant 3 in the mUQYBT. (mUQYBT = modified Upper Quarter Y-Balance Test)

Table 9: Mean distances (cm) per arm and arm length in the mUQYBT. (mUQYBT = modified Upper Quarter Y-Balance Test)

Table 10: Number of repetitions achieved in the CKCUEST. (CKCUEST = Closed Kinetic Chain Upper Extremity Speed Test)

Table 11: Number of repetitions achieved in the one-arm line hopping test. (Participant 3 did not do this test because of a ganglion in his right wrist.)

Table 12: QuickDASH module scores. (QuickDASH = Quick Disabilities of the Arm, Shoulder and Hand Questionnaire, PAM = Performing Arts Medicine)

Table 13: VLT scores of both participants. (VLT = Verbal Learning Test, PR = Percentile Rank, CI = Confidence Interval, s = seconds)

Table 14: NVLT scores of both participants. (NVLT = Non-Verbal Learning Test, PR = Percentile Rank, CI = Confidence Interval, s = seconds)

Table 15: TMT scores of both participants. (TMT = Trail Making Test, CI = Confidence Interval, s = seconds)

Table 16: TOL-F scores of both participants. (TOL-F = Tower of London – Freiburg Version, PR = Percentile Rank)

Table 17: INHIB scores of both participants. (INHIB = Response Inhibition, CI = Confidence Interval, s = seconds)

Table 18: Sample of raw data acquired through 3D Motion capture.

# Supplementary Figures


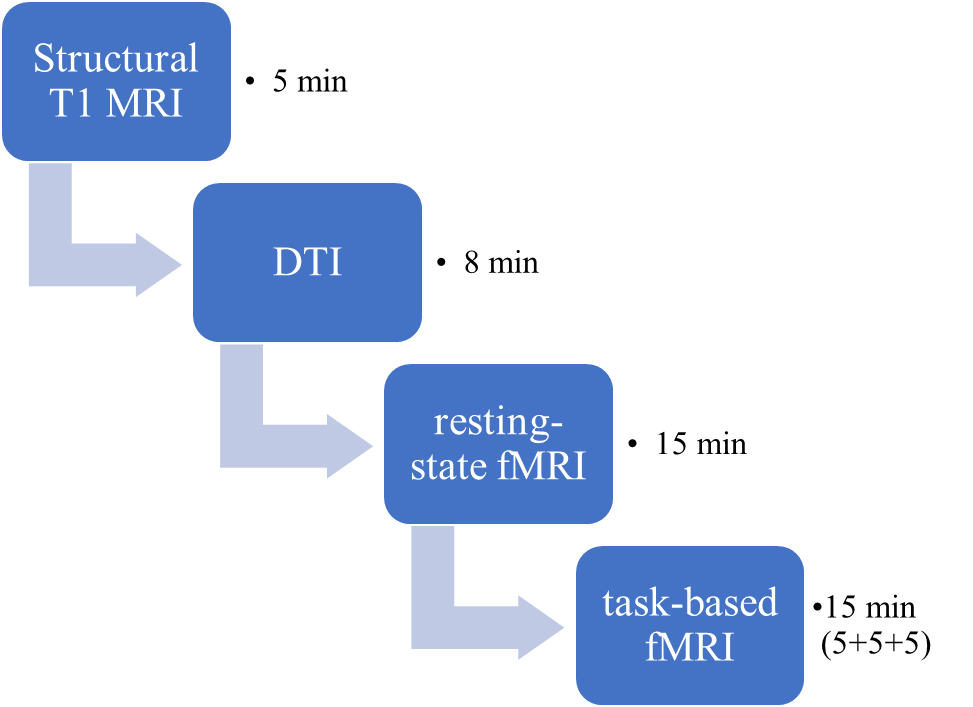


Figure 1: Medical imaging protocol with acquisition types and durations.


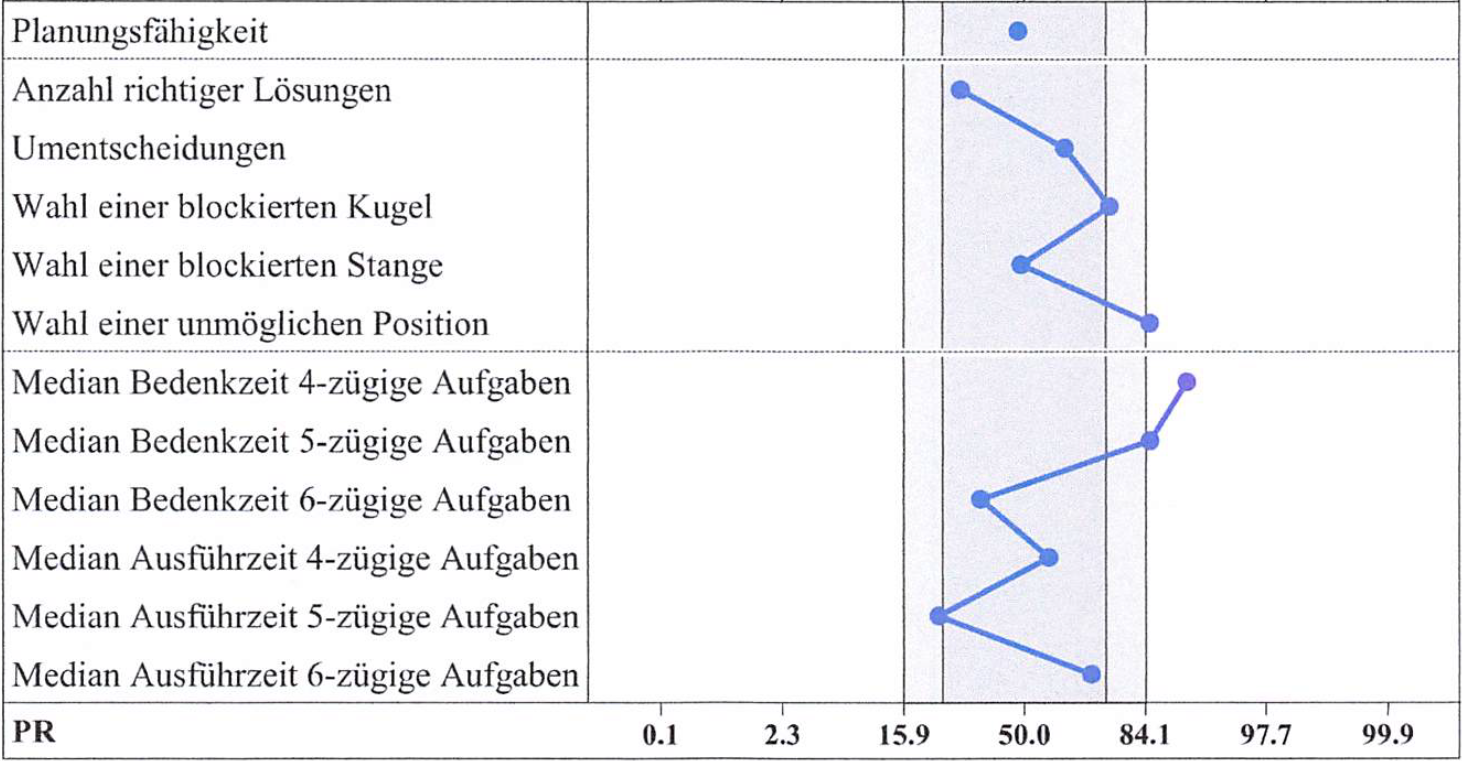


Figure 2: Percentile ranks achieved by participant 1 in the TOL-F. (TOL-F = Tower of London – Freiburg Version)


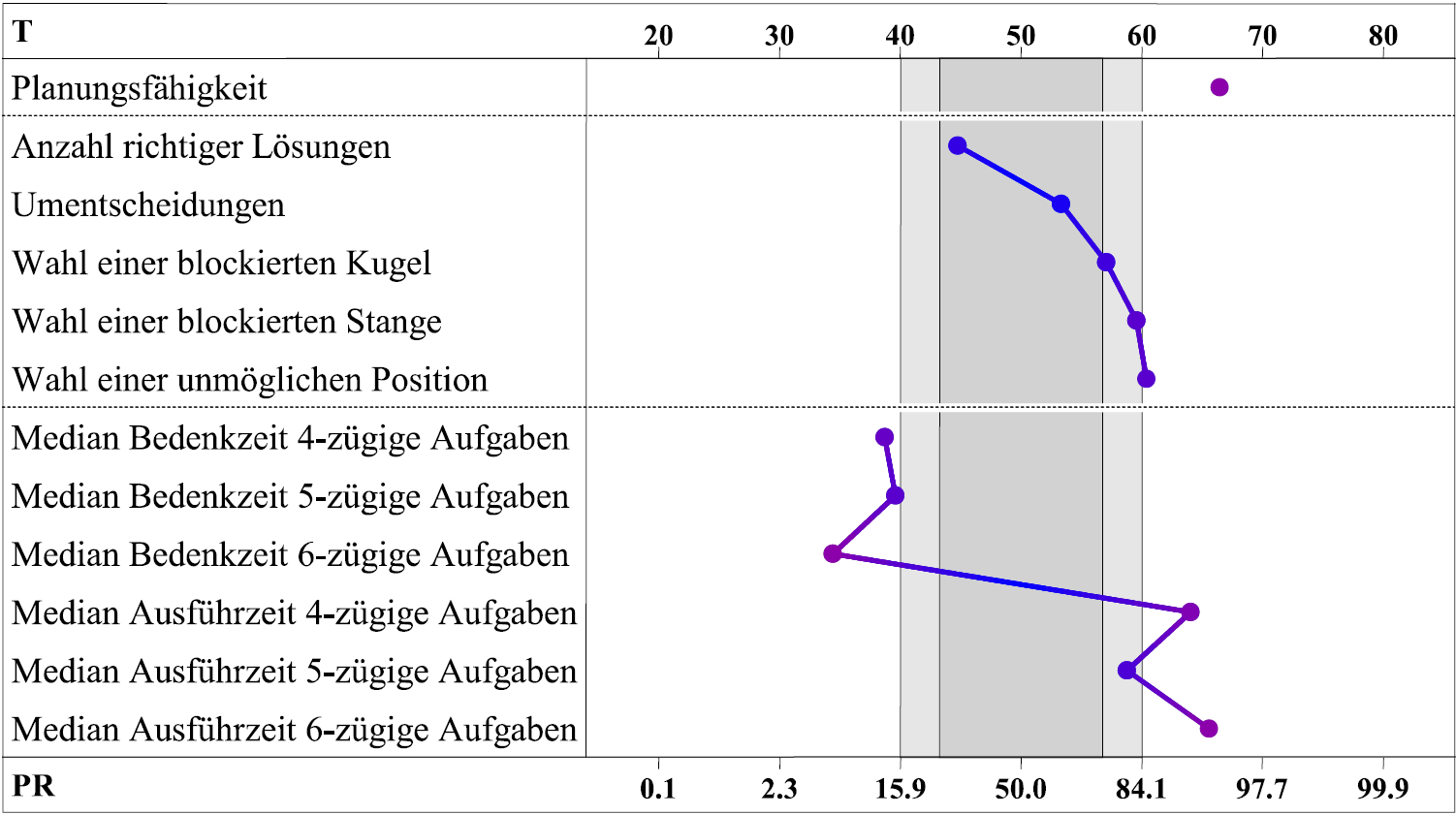


Figure 3: Percentile ranks achieved by participant 2 in the TOL-F. (TOL-F = Tower of London – Freiburg Version)


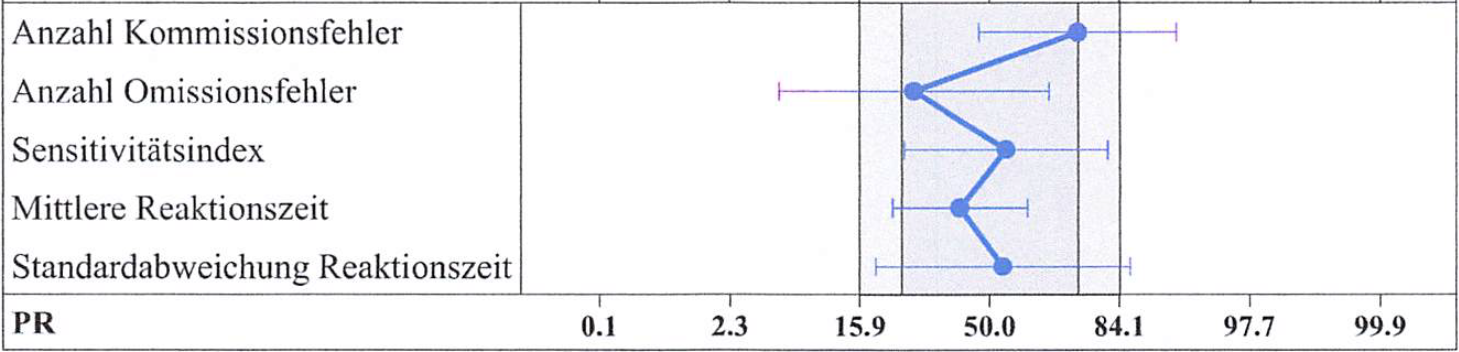


Figure 4: Percentile ranks with CIs achieved by participant 1 in the INHIB. (INHIB = Response Inhibition, CI = Confidence Interval)


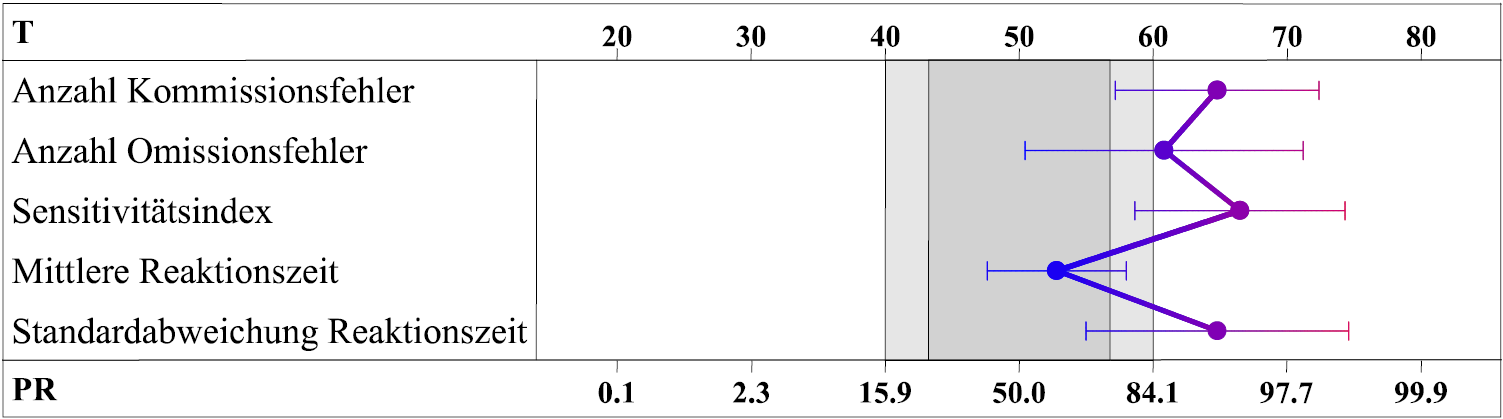


Figure 5: Percentile ranks with CIs achieved by participant 2 in the INHIB. (INHIB = Response Inhibition, CI = Confidence Interval)


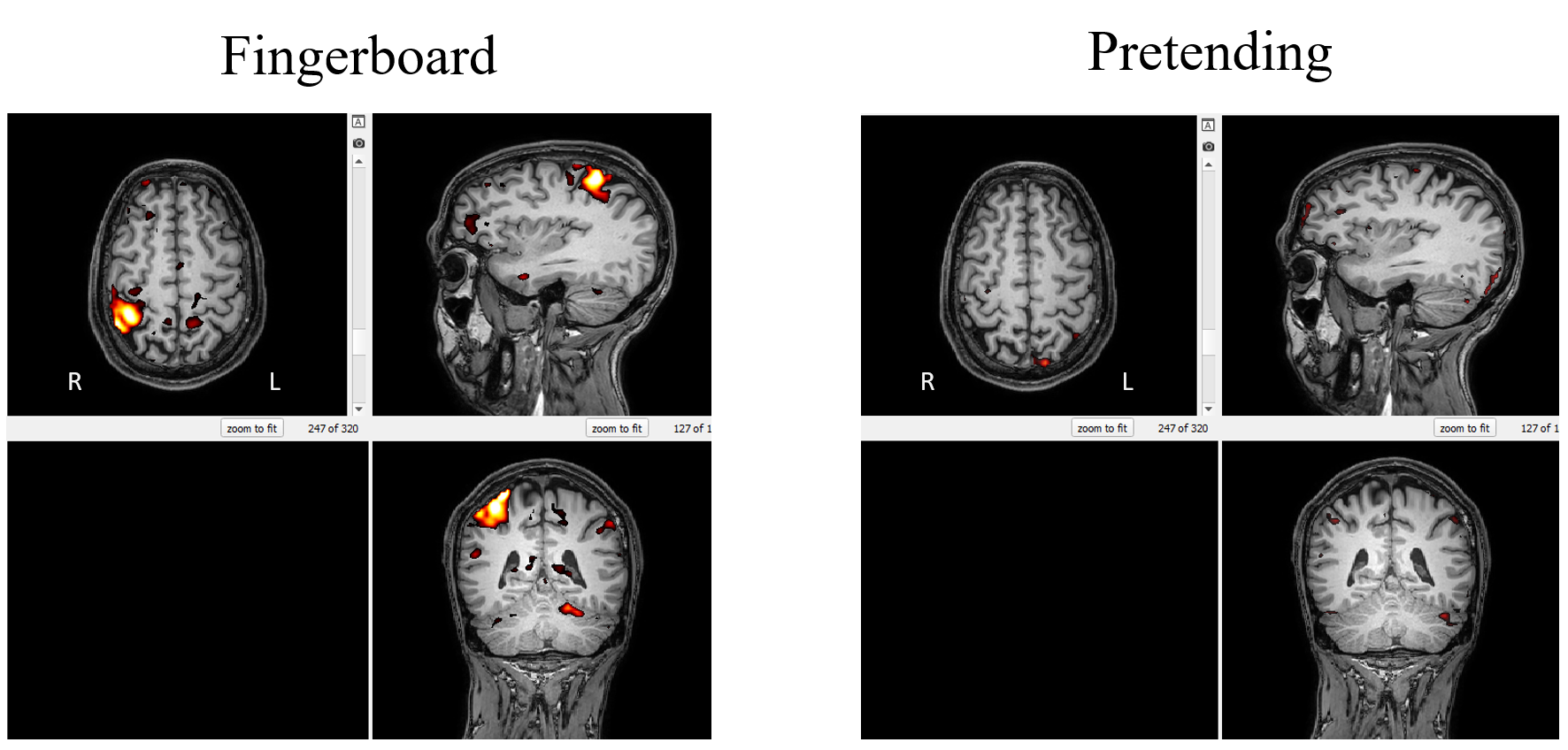


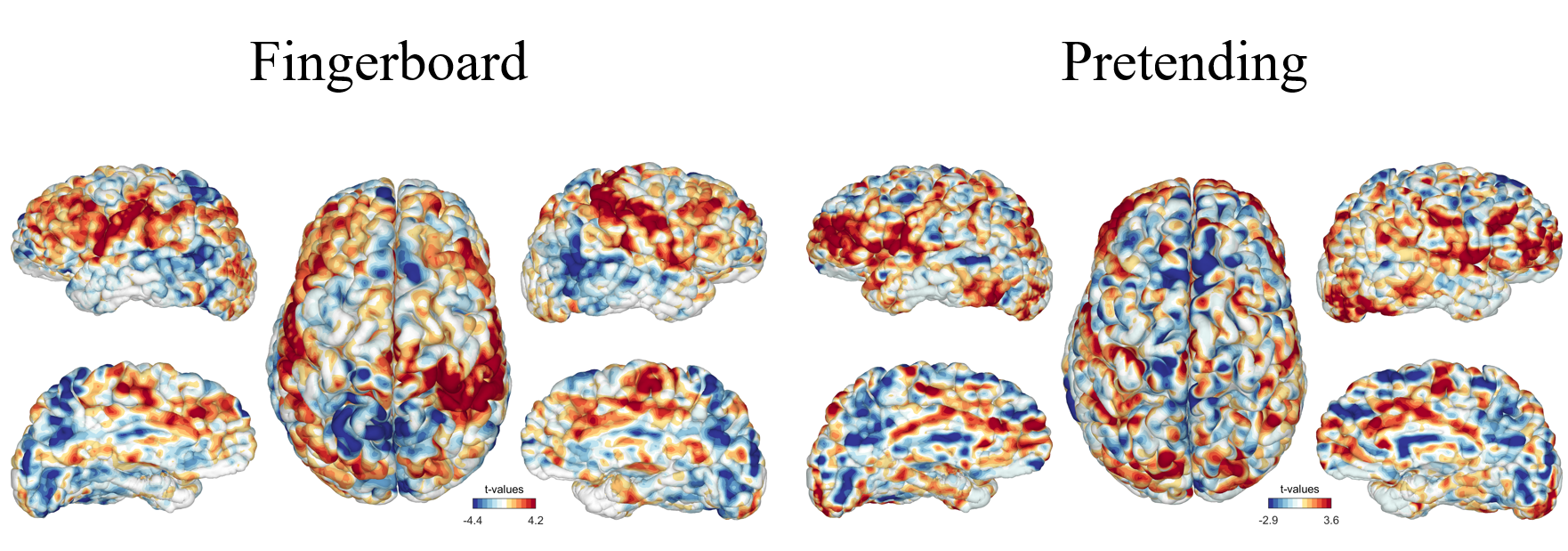


Figure 6: Task-based BOLD fMRI scans of participant 1.


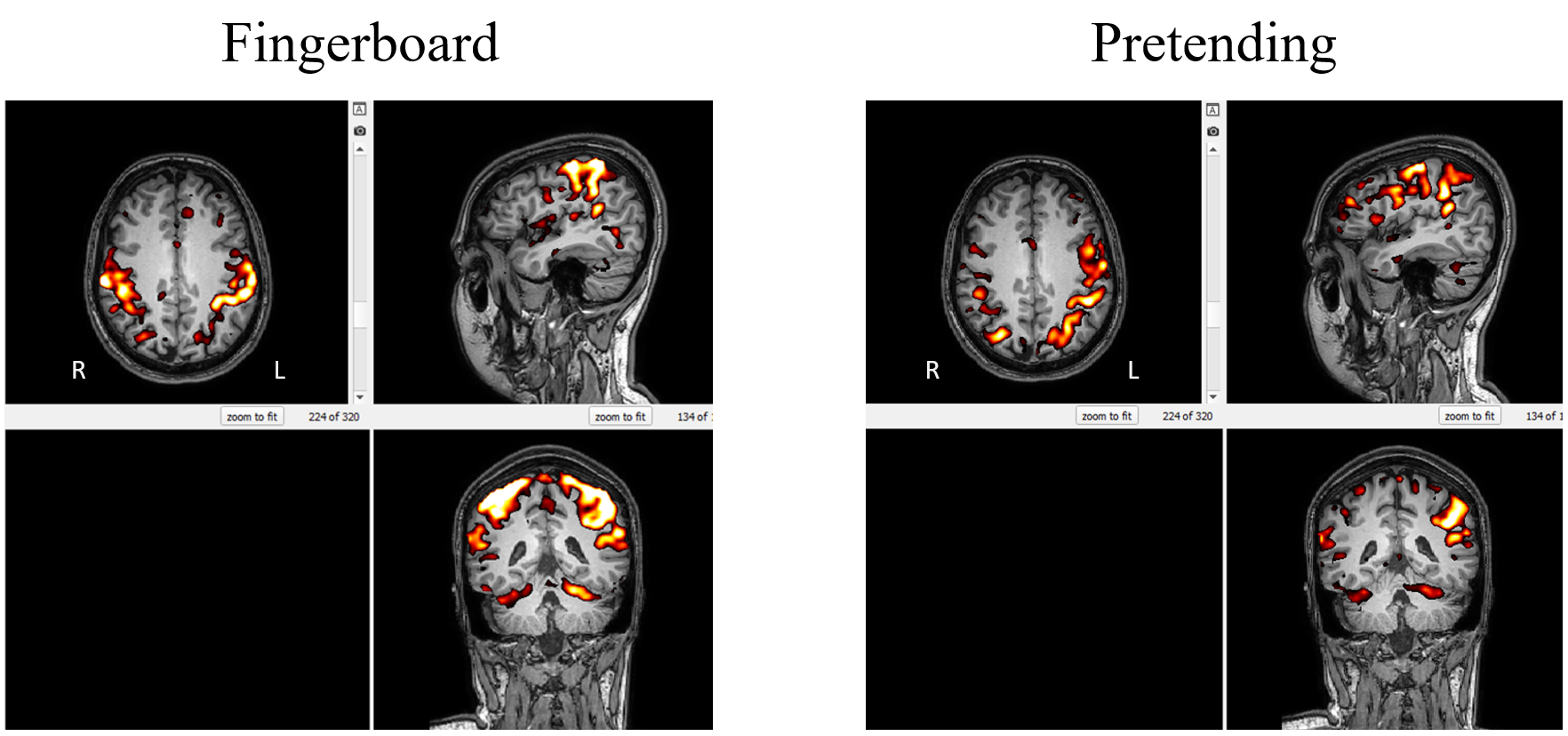


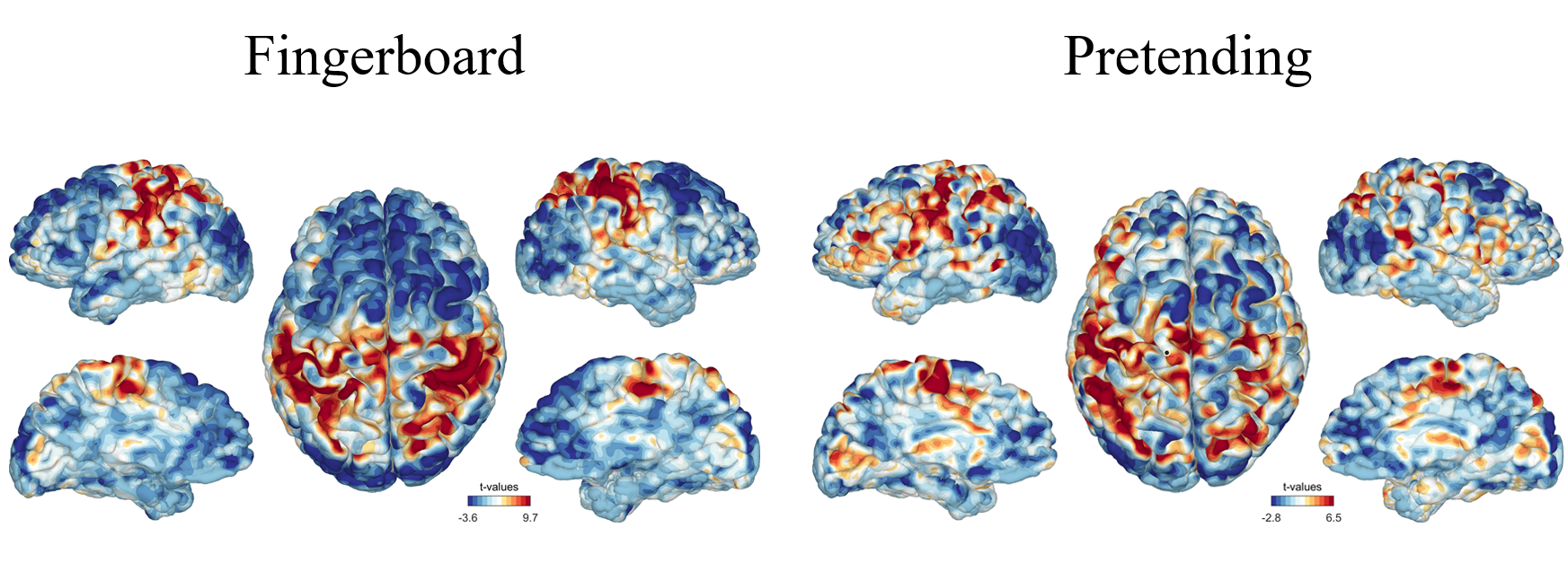


Figure 7: Task-based BOLD fMRI scans of participant 2.


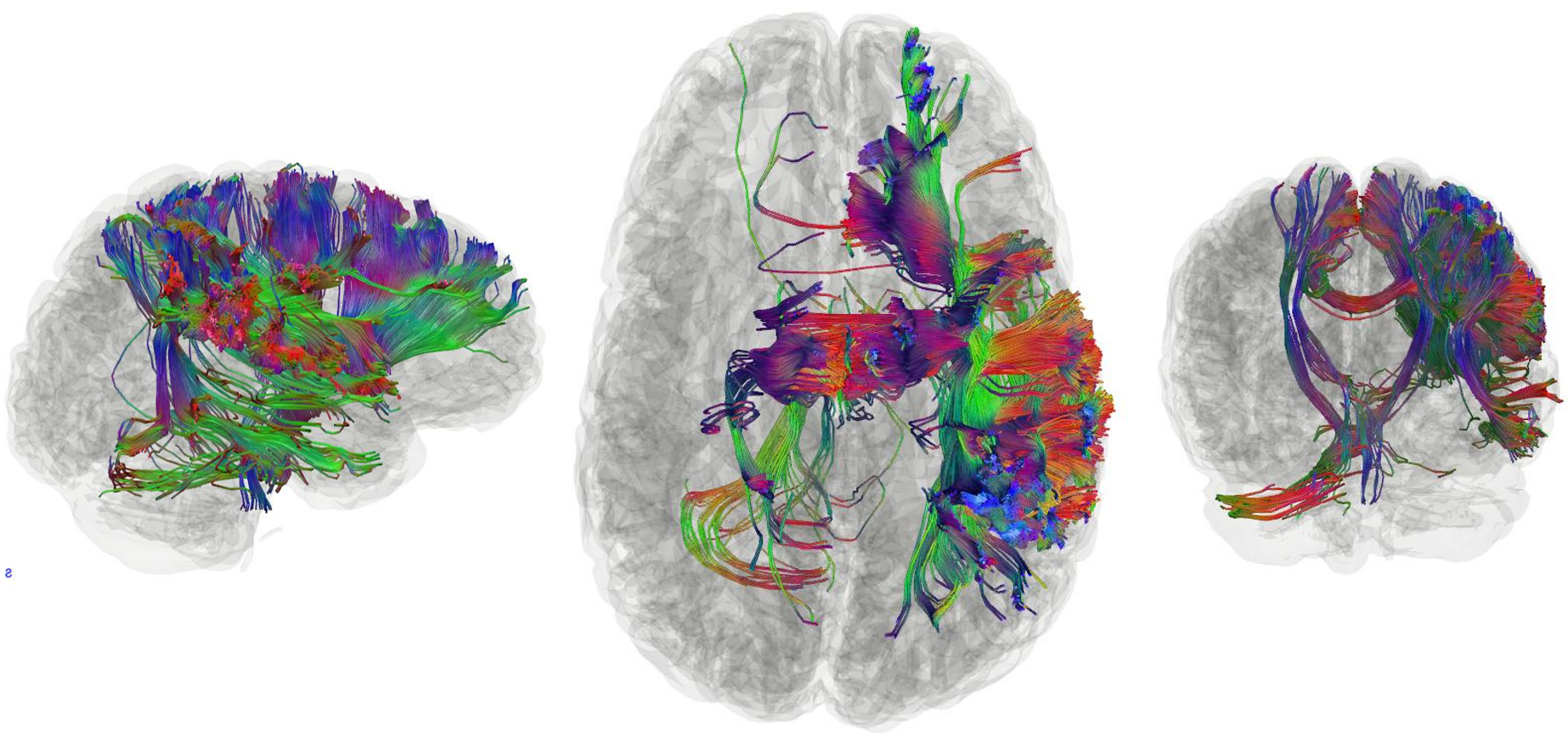


Figure 8: DTI Tractography of participant 1.


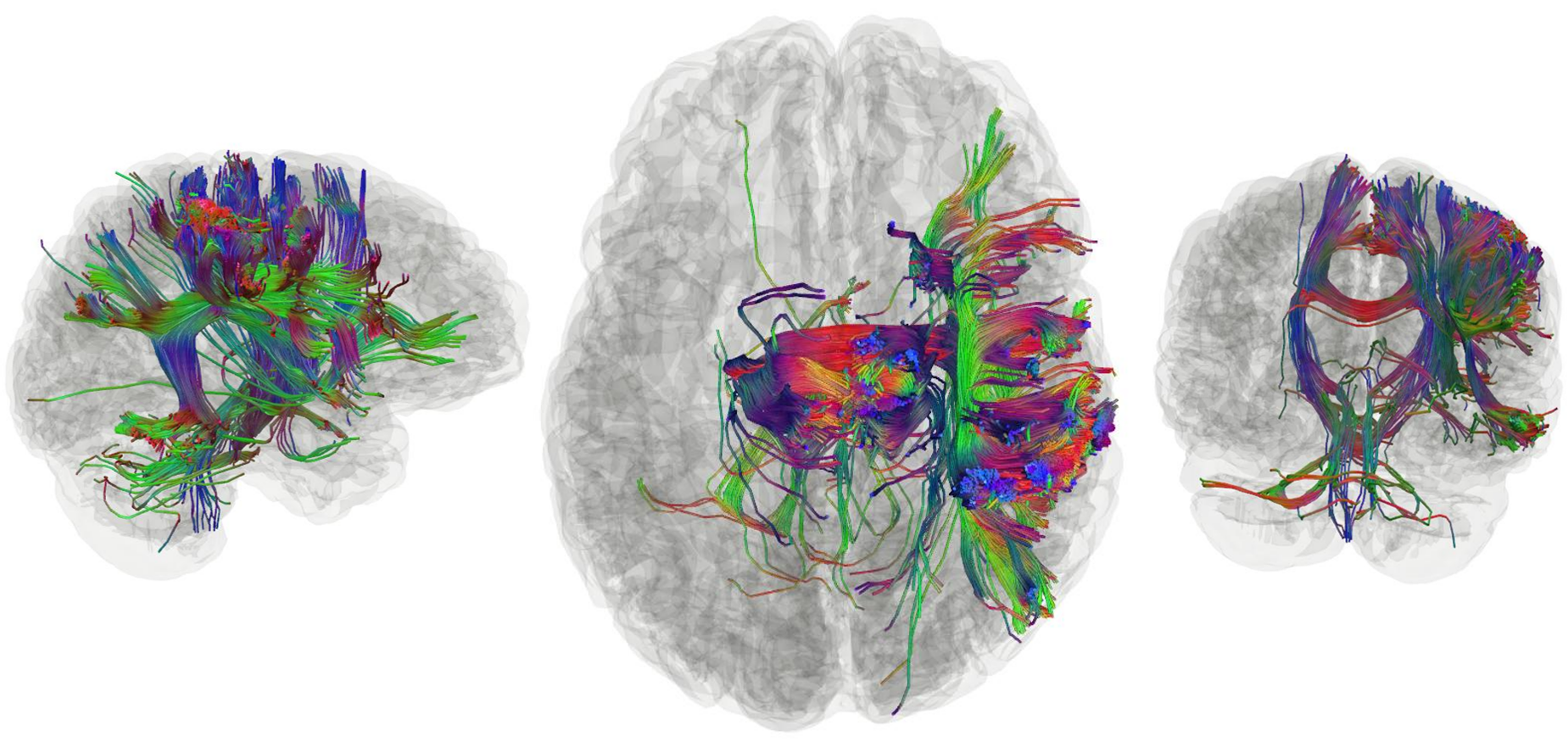


Figure 9: DTI Tractography of participant 2.

Figure 10: Wrist angle for two plays.

Figure 11: Elbow angle for two plays.

Figure 12: Shoulder angle for two plays.

Figure 13: Absolute deviation for the wrist-, elbow-, and shoulder angle.


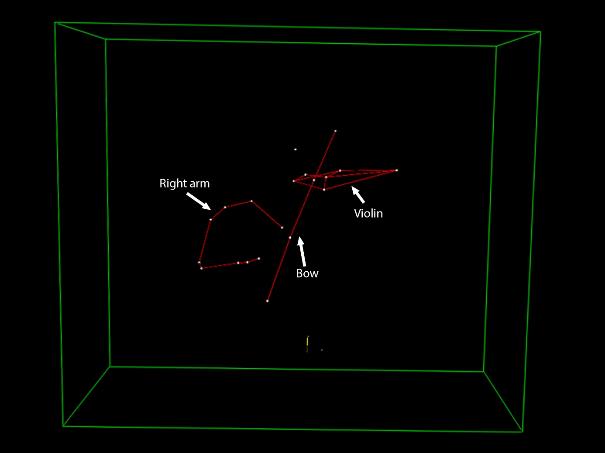


Figure 14: 3D motion capture model reconstruction (front view).

# Supplementary Tables

| **Participant ID** | **tDCS** | **Physio** | **Psychol. assessment** | **Medical Imaging** | **Motion** | **No. of trials** |
| --- | --- | --- | --- | --- | --- | --- |
| 1 |  |  |  | Yes |  | 1 |
| 2 |  |  |  | Yes |  | 1 |
| 3 |  |  |  |  | Yes | 1 |
| 4 | Yes | Yes |  |  |  | 2 |
| 5 |  | Yes |  |  |  | 1 |
| 6 | Yes | Yes | Yes |  |  | 3 |
| 7 |  | Yes | Yes |  |  | 2 |
| **No. of trials** | 2 | 4 | 2 | 2 | 1 |  |

Table 1: Pilot trials completed by each participant. (tDCS = transcranial Direct Current Stimulation)

| **Pilot trial** | **No. enrolled** | **No. completed** | **Time / participant** |
| --- | --- | --- | --- |
| tDCS with Halo Sport 2 | 2 | 2 | 100 |
| Physiotherapy | 3 | 3 | 110-125 |
| Coaching | 2 | 2 | 60 |
| Medical Imaging | 2 | 2 | 45 |
| Motion capture | 1 | 1 | 30 |

Table 2: Number of participants and total time (minutes) per pilot trial.

| **Issue** | **Participant 1** | **Participant 2** | **Total** |
| --- | --- | --- | --- |
| Connectivity | 2 | 1 | 3 |
| Moisture | 1 | 0 | 1 |
| Falling off | 1 | 0 | 1 |
| Tingling / Burning | 3 | 0 | 3 |

Table 3: Absolute frequency of issues with tDCS and the physiotherapeutic exercises.

|  | Yes | No |
| --- | --- | --- |
| When using Halo Sport 2, the device or software did not work as explained to me and/or I had trouble with the setup or handling of the device.  If yes, which: __________________________________________________ |  |  |
| When using Halo Sport 2, I experienced unfavourable/discomforting effects.  If yes, which: __________________________________________________ |  |  |
| During the therapy, I experienced unfavourable/discomforting effects that may be related to the use of Halo Sport 2.  If yes, which: __________________________________________________ |  |  |
| Unfavourable effects of Halo Sport 2 were an impairment for me in daily life.  If yes, which: __________________________________________________ |  |  |
| During the therapy, the symptoms of a disease I already had became more frequent or more severe.  If yes, which: __________________________________________________ |  |  |

Table 4: tDCS safety questionnaire.

| **Short statement** | **Participant 1** | **Participant 2** |
| --- | --- | --- |
| “I had trouble with setup/handling of the device.” | Yes | No |
| “I had adverse effects when using Halo Sport 2.” | Yes | No |
| “I had adverse effects during the therapy.” | Yes | No |
| “Adverse effects were an impairment in daily life.” | No | No |
| “The symptoms of a disease I had became worse.” | No | No |

Table 5: Answers to the "tDCS safety questionnaire" statements.

|  | **Lateral** | | **Superior** | | **Inferior** | |
| --- | --- | --- | --- | --- | --- | --- |
|  | **right arm** | **left arm** | **right arm** | **left arm** | **right arm** | **left arm** |
| **First try** | 103 | 95 | 68 | 59 | 101 | 97 |
| **Second try** | 104 | 98 | 63 | 62 | 103 | 101 |
| **Third try** | 103 | 95 | 61 | 65 | 103 | 107 |
| **Mean ± SD** | 103 | 96 | 64 | 62 | 102 | 102 |

Table 6: Distances (cm) achieved by participant 1 in the mUQYBT. (mUQYBT = modified Upper Quarter Y-Balance Test)

|  | **Lateral** | | **Superior** | | **Inferior** | |
| --- | --- | --- | --- | --- | --- | --- |
|  | **right arm** | **left arm** | **right arm** | **left arm** | **right arm** | **left arm** |
| **First try** | 95 | 93 | 65 | 62 | 74 | 74 |
| **Second try** | 102 | 101 | 58 | 63 | 72 | 72 |
| **Third try** | 107 | 103 | 62 | 64 | 75 | 75 |
| **Mean ± SD** | 101 | 99 | 62 | 63 | 74 | 74 |

Table 7: Distances (cm) achieved by participant 2 in the mUQYBT. (mUQYBT = modified Upper Quarter Y-Balance Test)

|  | **Lateral** | | **Superior** | | **Inferior** | |
| --- | --- | --- | --- | --- | --- | --- |
|  | **right arm** | **left arm** | **right arm** | **left arm** | **right arm** | **left arm** |
| **First try** | 111 | 97 | 59 | 55 | 97 | 88 |
| **Second try** | 111 | 106 | 60 | 54 | 101 | 82 |
| **Third try** | 110 | 107 | 56 | 82 | 98 | 80 |
| **Mean** | 111 | 103 | 58 | 64 | 99 | 83 |

Table 8: Distances (cm) achieved by participant 3 in the mUQYBT. (mUQYBT = modified Upper Quarter Y-Balance Test)

|  | **Right arm** | **Left arm** | **Arm length** |
| --- | --- | --- | --- |
| **Participant 1** | 107 | 103 | 84 |
| **Participant 2** | 88 | 87 | 91 |
| **Participant 3** | 106 | 99 | 97 |

Table 9: Mean distances (cm) per arm and arm length in the mUQYBT. (mUQYBT = modified Upper Quarter Y-Balance Test)

|  | **Participant 1** | **Participant 2** | **Participant 3** |
| --- | --- | --- | --- |
| **First try** | 13 | 8 | 10 |
| **Second try** | 16 | 9 | 11 |
| **Third try** | 18 | 10 | 12 |
| **Mean** | 16 | 9 | 11 |

Table 10: Number of repetitions achieved in the CKCUEST. (CKCUEST = Closed Kinetic Chain Upper Extremity Speed Test)

|  | **Participant 1** | **Participant 2** | **Participant 3** |
| --- | --- | --- | --- |
| **Right arm** | 41 | 25 | n.a. |
| **Left arm** | 52 | 22 | n.a. |
| **Mean** | 47 | 10 | n.a. |

Table 11: Number of repetitions achieved in the one-arm line hopping test. (Participant 3 did not do this test because of a ganglion in his right wrist.)

|  | **Participant 1** | **Participant 2** | **Participant 3** |
| --- | --- | --- | --- |
| **Main module score** | 9.1 | 0 | 0 |
| **Work module score** | 6.3 | 0 | 12.5 |
| **PAM module score** | 6.3 | 0 | 12.5 |

Table 12: QuickDASH module scores. (QuickDASH = Quick Disabilities of the Arm, Shoulder and Hand Questionnaire, PAM = Performing Arts Medicine)

|  | **Participant 1** | | **Participant 2** | |
| --- | --- | --- | --- | --- |
|  | **Sum** | **PR (95% CI)** | **Sum** | **PR (95% CI)** |
| **Correct YES-responses** | 40 | 89 (73-96) | 39 | 66 (42-84) |
| **Incorrect YES-responses** | 21 | 10 (2-27) | 16 | 24 (8-50) |
| **Difference of correct minus incorrect YES-responses** | 19 | 21 (8-42) | 23 | 33 (16-58) |
| **Median of the reaction time for correct YES-responses (s)** | 0.65 | | 0.97 | |
| **Median of the reaction time for incorrect YES-responses (s)** | 0.84 | | 1.49 | |
| **Total working time (min:sec)** | 01:50 | | 02:08 | |
| **Frainlness index** | 0.00 | | 0.06 | |

Table 13: VLT scores of both participants. (VLT = Verbal Learning Test, PR = Percentile Rank, CI = Confidence Interval, s = seconds)

|  | **Participant 1** | | **Participant 2** | |
| --- | --- | --- | --- | --- |
|  | **Sum** | **PR (95% CI)** | **Sum** | **PR (95% CI)** |
| **Correct YES-responses** | 33 | 49 (24-76) | 32 | 43 (18-69) |
| **Incorrect YES-responses** | 8 | 72 (50-88) | 10 | 66 (42-84) |
| **Difference of correct minus incorrect YES-responses** | 25 | 82 (54-96) | 22 | 71 (42-92) |
| **Total working time (min:sec)** | 02:00 | | 04:11 | |
| **Frailness index** | 0.25 | | 0.13 | |

Table 14: NVLT scores of both participants. (NVLT = Non-Verbal Learning Test, PR = Percentile Rank, CI = Confidence Interval, s = seconds)

|  | **Participant 1** | | **Participant 2** | |
| --- | --- | --- | --- | --- |
|  | **Value** | **PR (95% CI)** | **Value** | **PR (95% CI)** |
| **Working time part A (s)** | 17.34 | 20 (10-38) | 9.08 | 100 (99-100) |
| **Working time part B (s)** | 20.68 | 63 (38-82) | 15.30 | 97 (90-99) |
| **Working time part A corrected (s)** | 17.34 | 19 | 9.08 | 100 |
| **Working time part B corrected (s)** | 19.67 | 69 | 15.30 | 96 |
| **Errors part A** | 0 | 52 | 0 | 52 |
| **Errors part B** | 1 | 31 | 0 | 72 |
| **Time difference B-A (s)** | 3.34 | 86 | 6.22 | 62 |
| **Time quotient B/A** | 1.19 | 89 (66-98) | 1.69 | 30 (10-62) |

Table 15: TMT scores of both participants. (TMT = Trail Making Test, CI = Confidence Interval, s = seconds)

|  | **Participant 1** | | **Participant 2** | |
| --- | --- | --- | --- | --- |
|  | **Value** | **PR** | **Value** | **PR** |
| **Planning ability** | 16 | 48 | 21 | 95 |
| **Number of correctly solved items** | 23 | 30 | 23 | 30 |
| **Number of reversed decisions** | 4 | 63 | 4 | 63 |
| **Number of blocked balls selected** | 0 | 76 | 0 | 76 |
| **Number of blocked rods selected** | 2 | 49 | 0 | 83 |
| **Number of impossible positions selected** | 0 | 85 | 0 | 85 |
| **Median planning time of 4-action tasks** | 2.5 | 91 | 7.6 | 13 |
| **Median planning time of 5-action tasks** | 4.2 | 85 | 12.4 | 15 |
| **Median planning time of 6-action tasks** | 9.5 | 36 | 18.0 | 6 |
| **Median execution time of 4-action tasks** | 4.9 | 58 | 3.9 | 92 |
| **Median execution time of 5-action tasks** | 12.2 | 24 | 5.8 | 81 |
| **Median execution time of 6-action tasks** | 9.5 | 71 | 6.5 | 94 |

Table 16: TOL-F scores of both participants. (TOL-F = Tower of London – Freiburg Version, PR = Percentile Rank)

|  | **Participant 1** | | **Participant 2** | |
| --- | --- | --- | --- | --- |
|  | **Value** | **PR (95% CI)** | **Value** | **PR (95% CI)** |
| **Commission errors** | 5 | 75 (46-93) | 2 | 93 (76-99) |
| **Omission errors** | 3 | 28 (5-66) | 0 | 86 (54-98) |
| **Sensitivity index** | 3.430 | 55 (24-82) | 4.310 | 95 (79-99) |
| **Mean reaction time (s)** | 0.272 | 41 (24-62) | 0.253 | 61 (42-79) |
| **Standard deviation of mean reaction time (s)** | 0.078 | 54 (18-86) | 0.042 | 93 (69-99) |
| **Total working time** | 01:51 | | 01:46 | |

Table 17: INHIB scores of both participants. (INHIB = Response Inhibition, CI = Confidence Interval, s = seconds)

| **Wrist angle (°)** | | | |
| --- | --- | --- | --- |
| **Time (s)** | **Play 1** | **Play 2** | **Mean absolute deviation (°)** |
| 0 | 0.053 | 0.034 | 0.019 |
| 0.008 | 0.002 | 0.02 | 0.018 |
| 0.017 | -0.05 | -0.07 | 0.016 |
| 0.025 | -0.03 | -0.13 | 0.095 |
| 0.033 | -0.03 | -0.11 | 0.082 |
| 0.042 | -0.04 | -0.09 | 0.047 |
| 0.05 | -0.06 | -0.15 | 0.093 |
| 0.058 | -0.06 | -0.16 | 0.094 |
| 0.067 | -0.08 | -0.16 | 0.076 |
| 0.075 | -0.12 | -0.19 | 0.071 |
| 0.083 | -0.15 | -0.21 | 0.063 |
| 0.092 | -0.15 | -0.23 | 0.083 |
| 0.1 | -0.19 | -0.29 | 0.106 |
| 0.108 | -0.23 | -0.33 | 0.097 |
| 0.117 | -0.26 | -0.32 | 0.053 |
| 0.125 | -0.31 | -0.25 | 0.057 |
| 0.133 | -0.37 | -0.26 | 0.105 |
| 0.142 | -0.41 | -0.24 | 0.169 |
| 0.15 | -0.49 | -0.27 | 0.22 |

Table 18: Sample of raw data acquired through 3D Motion capture.
